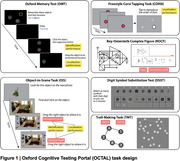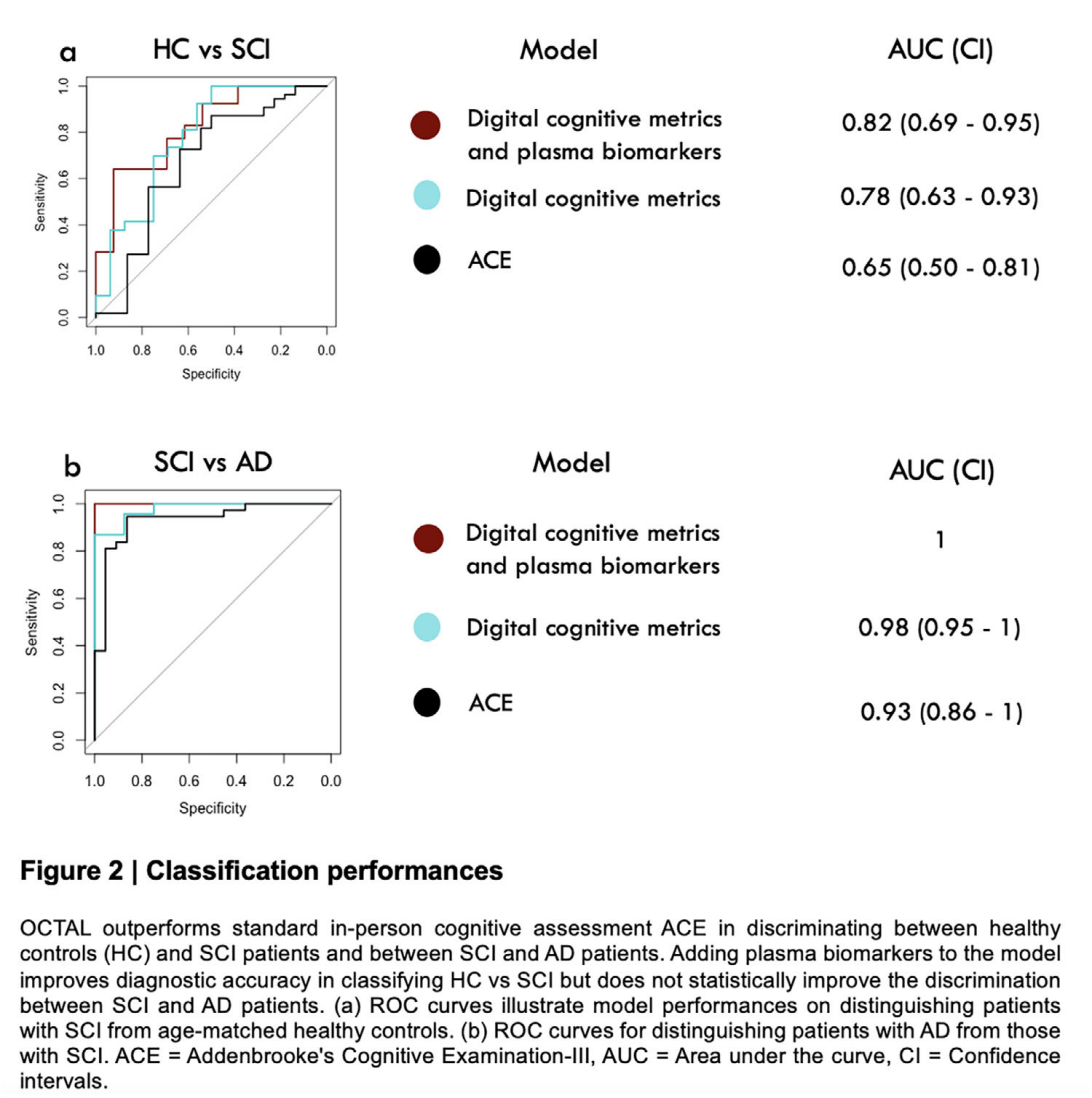# Diagnostic performance of online cognitive assessment and plasma biomarkers in Alzheimer’s Disease and Subjective Cognitive Impairment using the Oxford Cognitive Testing Portal

**DOI:** 10.1002/alz.094324

**Published:** 2025-01-09

**Authors:** Sofia Toniolo, Anna Scholcz, Benazir Amein, Akke Ganse‐Dumrath, Sian Thompson, Sanjay Manohar, Henrik Zetterberg, Masud Husain, Sijia Zhao

**Affiliations:** ^1^ University of Oxford, Oxford UK; ^2^ Oxford University Hospitals NHS Foundation Trust, Oxford UK; ^3^ Hong Kong Center for Neurodegenerative Diseases, Hong Kong China

## Abstract

**Background:**

Plasma biomarkers have emerged as a promising tool to detect the presence of Alzheimer’s disease (AD) when cognitive symptoms have not yet emerged. However, there is also a pressing need to detect and track subtle cognitive change at the preclinical stage of AD for population screening purposes and to monitor disease progression at scale. A potential solution is remote cognitive assessment, yet it is still not extensively employed.

**Method:**

114 participants (37 AD, 22 subjective cognitive impairment (SCI) patients and 55 age‐matched controls) were recruited from the Oxford Centre for Cognitive Disorders. They were tested on a newly developed, fully remote online cognitive assessment tool, Oxford Cognitive Testing Portal (OCTAL), which hosts a wide range of validated cognitive tasks, such as the Rey‐Osterrieth Complex Figure (ROCF), Trail Making Task (TMT), Digital Symbol Substitution task (DSST)), Corsi Block Task (CORSI), as well as novel visual short‐term memory (Oxford Memory task) and visual long‐term memory (Object‐in‐Scene task) tasks (Figure 1). All participants underwent standard in‐person cognitive testing, i.e. Addenbrooke’s Cognitive Examination‐III (ACE). Plasma p‐tau181, GFAP, NFL, Aß42/40 ratio were also measured. Logistic regression was used for group classification.

**Result:**

Performance on OCTAL was able to discriminate between SCI from healthy controls with an AUC of 0.78 (Figure 2a), statistically outperforming standard neuropsychological testing (ACE), which had an AUC of 0.65. Combining plasma biomarkers to OCTAL further increased diagnostic accuracy, reaching an AUC of 0.82 in group classification. OCTAL also outperformed ACE in distinguishing individuals with AD from SCI (Figure 2b), where adding plasma biomarkers to performance at OCTAL achieved a perfect separation (AUC of 1) between the groups.

**Conclusion:**

These findings demonstrate the potential of OCTAL for widespread, cost‐effective cognitive testing. They also emphasise the utility of combining plasma biomarkers and digital cognitive tests to improve diagnostic accuracy across different stages of the disease. These accessible tools could pave the way to more scalable protocols for screening, stratification and monitoring of patients with preclinical AD.